# Supplementary material for: A Common Target of Nitrite and Nitric Oxide for Respiration Inhibition in Bacteria
Source: Int J Mol Sci. 2022 Nov 10;23(22):13841. doi: 10.3390/ijms232213841 (PMC9697910; doi:10.3390/ijms232213841)
Supplement: Supplementary file 1 [file ijms-23-13841-s001.zip › ijms-2030340-supplementary.pdf]

Supplemental Figures for

A common target of nitrite and nitric oxide for respiration inhibition in bacteria

Wei Wang, Jiahao Wang, Xue Feng, and Haichun Gao*

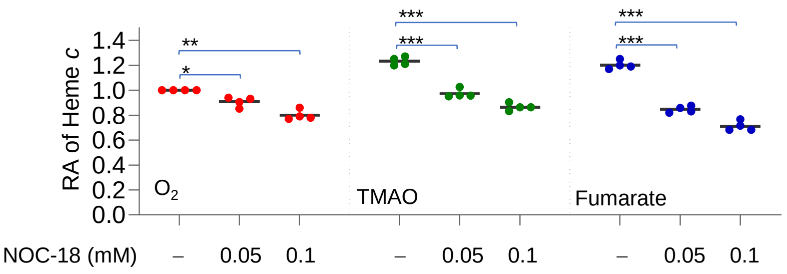

Figure S1. The cyt *c* content in cells grown with NOC-18. WT cells grown to the early stationary phase with NOC-18 at varying concentrations were collected for heme *c* quantification. The data were first adjusted to the protein levels of samples, and then the averaged heme *c* levels of the mutants were normalized to that in WT, which was set to 1, giving to relative abundance (RA). Asterisks indicate statistically significant difference of the values compared (*, $P < 0.05$; **, $P < 0.01$; ***, $P < 0.001$).

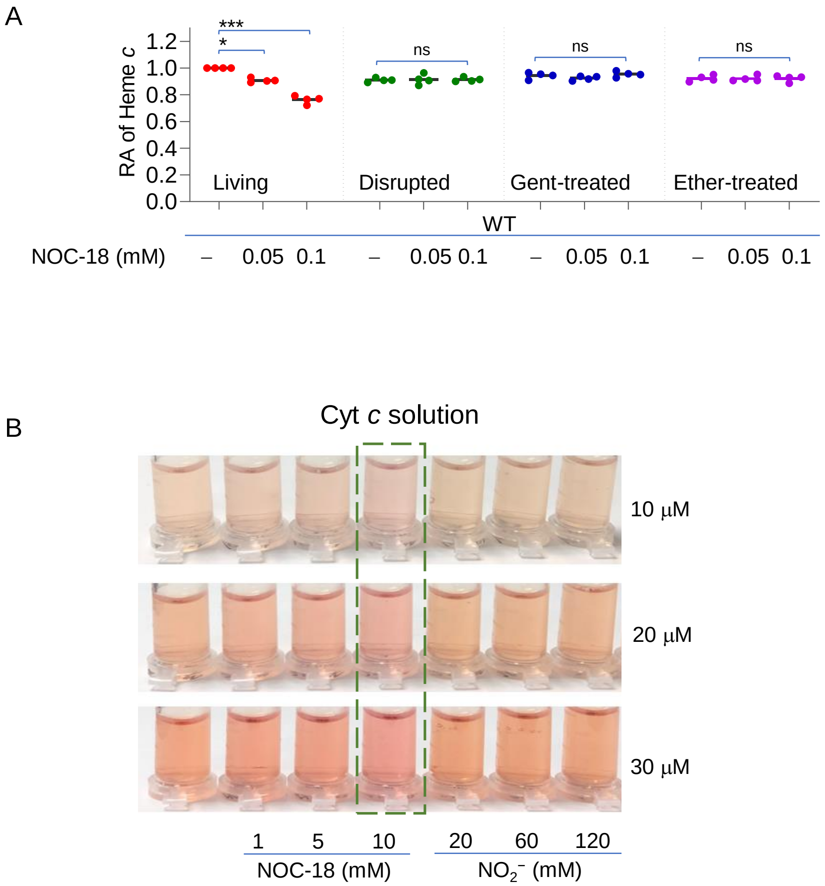

Figure S2. Impacts of nitrite/NO on cyt *c*. **A** Heme *c* levels. Cells at the early stationary phase disrupted by sonication, or treated by gentamycin and by ether were incubated with NOC-18 at varying concentrations for 5 hours. Quantification of heme *c* was performed and the data were processed as described in Fig. 1b. Asterisks indicate statistically significant difference of the values compared (n = 4; ns, not significant; *, $P < 0.05$; ***, $P < 0.001$). **B** Impacts of nitrite/NO on cyt *c* solution. The bovine hear cyt *c* solutions of indicated concentrations were treated with NOC-18 and nitrite for 5 hours. NOC-18 at 10 mM evidently turned the solution more pinkish.

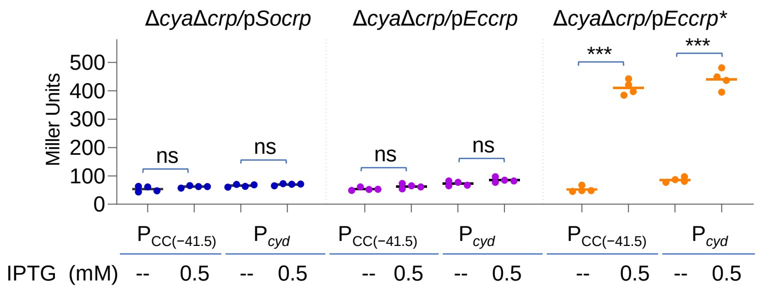

Figure S3. *Ec*Crp$^{T128L-S129I}$ (encoded by *Eccrp**) is independent of cAMP in *S. oneidensis*. Production of *So*Crp, *Ec*Crp, and *Ec*Crp$^{T128L-S129I}$ was driven by IPTG-inducible promoter in *S. oneidensis crp* and *crp-cya* mutants. $P_{CC(-41.5)}$ and P_{cyd} are promoters that are directly controlled by *Ec*Crp and *So*Crp, respectively. Promoter activity in cells grown to the early stationary phase was determined by *lacZ* reporters. Asterisks indicate statistically significant difference of the values compared ($n = 4$; ns, not significant; ***, $P < 0.001$).

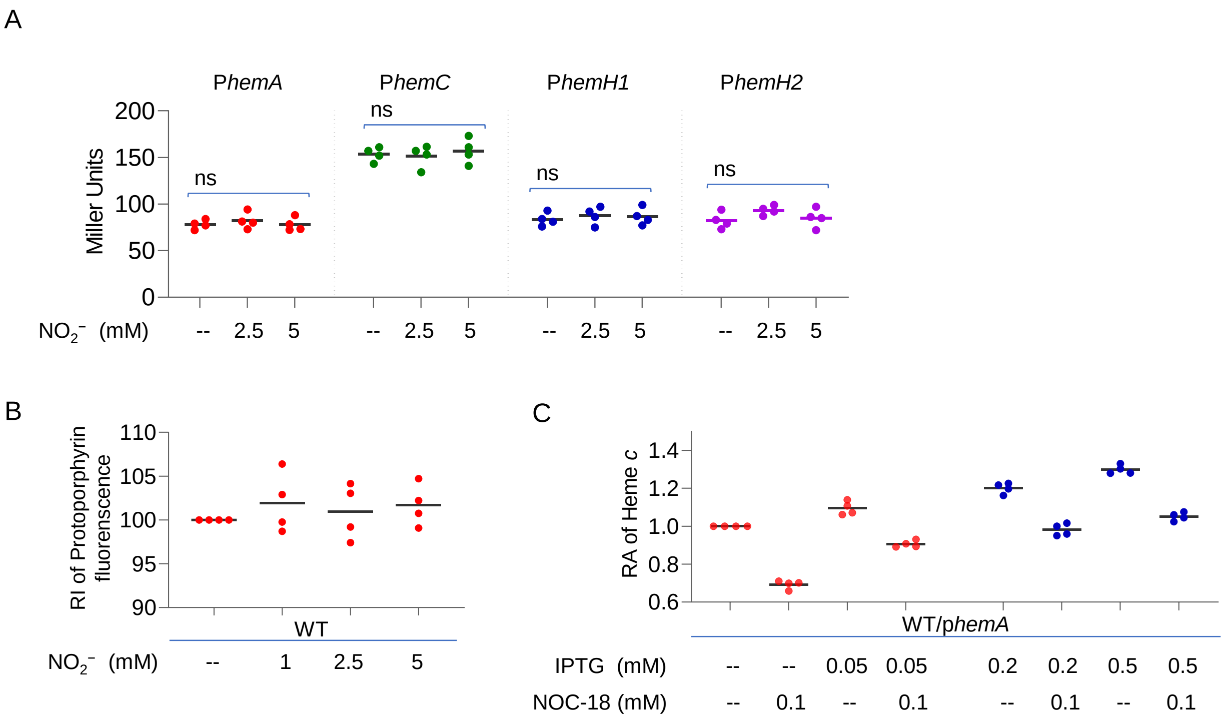

Figure S4. Heme antagonizes inhibitory effects of nitrite/NO on the cyt *c* content. **A** Impacts of nitrite on expression of key genes in the heme synthesis pathway. Asterisks indicate statistically significant difference of the values compared (*n* = 4; ns, not significant). Similar results were obtained from 0.1 mM NOC-18. **B** Heme quantification by pyridine hemochromogen assay. Relative intensity (RI) was given by setting WT values to 100%. **C** Heme antagonizes inhibitory effects of NO on the cyt *c* content. Heme *c* levels were quantified and presented as relative abundance (RA). HemA is the enzyme catalyzing the rating limiting step in heme biosynthesis. Production of HemA was driven by IPTG-inducible promoter in *S. oneidensis*. Asterisks indicate statistically significant difference of the values compared (*n* = 4; ns, not significant).

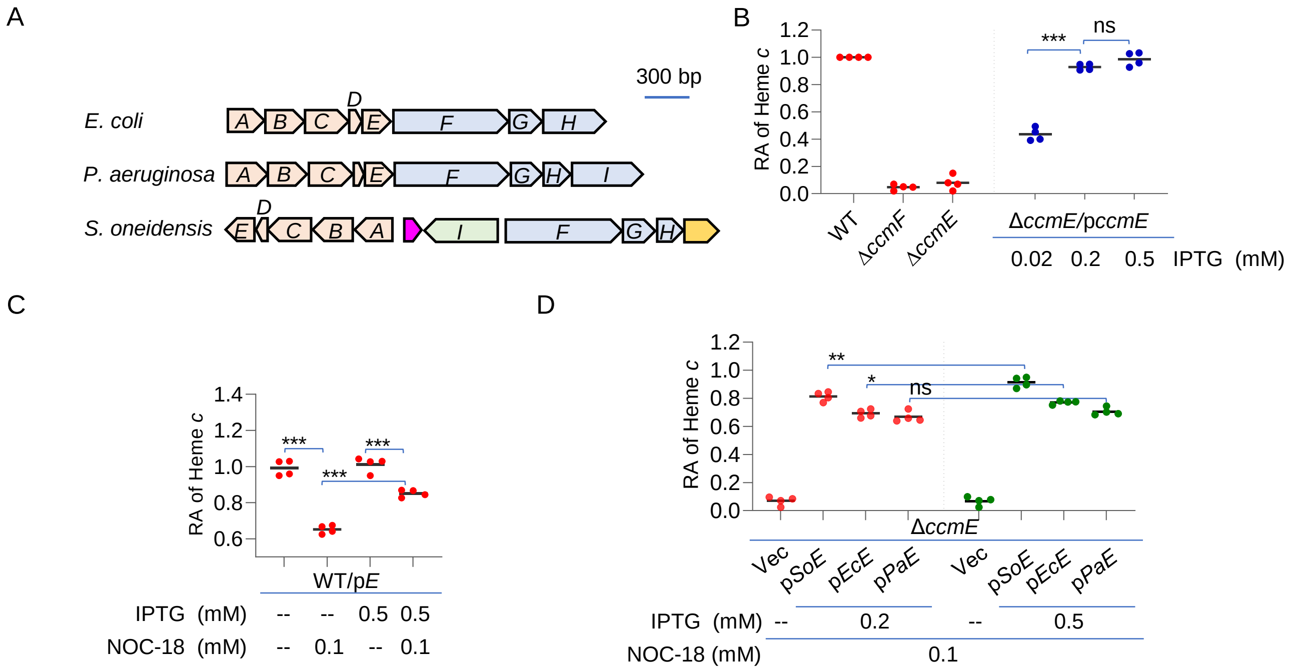

Figure S5. CcmE is a likely target of nitrite/NO in cyts *c* production. **A** Organization of the *ccm* genes in γ-proteobacteria. In *S. oneidensis*, *ccm* genes are clustered but constitute multiple operons. Two common gene arrangements are shown, represented by *P. aeruginosa* in which CcmH and CcmI are separate from each other and by *E. coli* whose CcmH is a fusion protein between CcmH and the C-terminal portion of CcmI as found in other bacteria. Genes are drawn to scale. **B** Heme *c* levels of the *ccmE* mutant compared to positive control (WT) and negative control (Δ*ccmF*) strains. Heme *c* levels in the *ccmE* mutant producing CcmE driven by IPTG-inducible promoter with IPTG at varying levels. **C** Heme *c* levels in WT overproducing CcmE without or with 0.1 mM NOC-18. **D** Heme *c* levels in the *ccmE* mutant overproducing *S. oneidensis*, *E. coli* and *P. aeruginosa* CcmE driven by IPTG-inducible promoter without or with 0.1 mM NOC-18. Asterisks indicate statistically significant difference of the values compared ($n = 4$; ns, not significant; *, $P < 0.05$; **, $P < 0.01$; ***, $P < 0.001$).

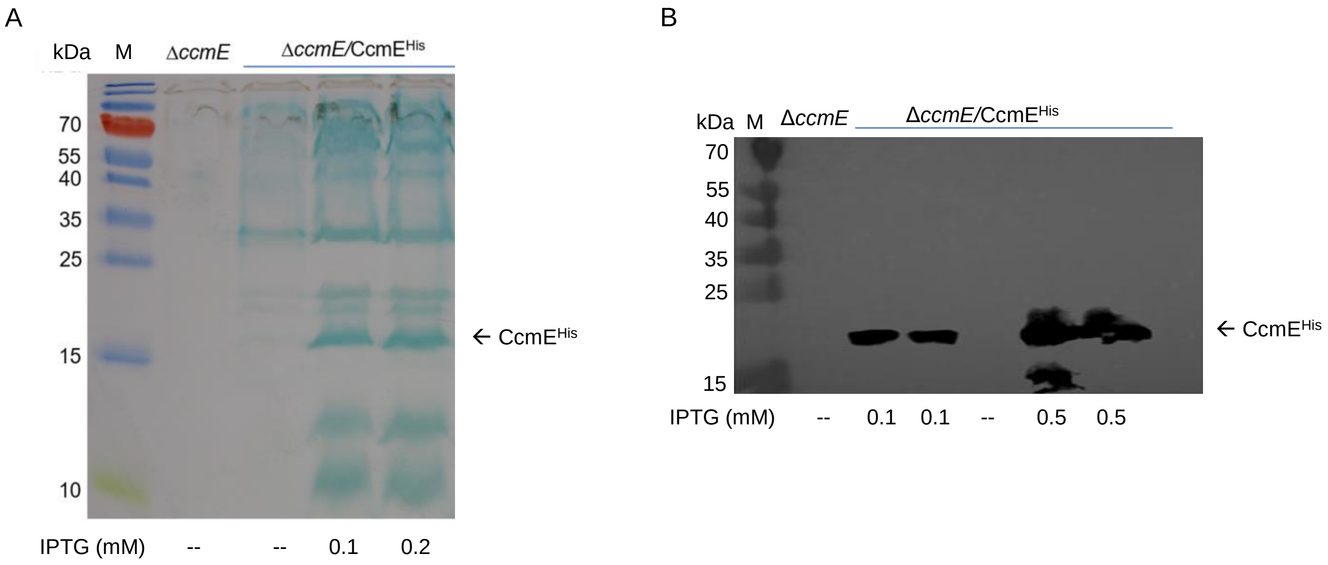

Figure S6. His-Tagged CcmE (CcmE^{His}) recombinant proteins are functional. **A** Heme staining. The defect of the *ccmE* mutant (Δ*ccmE*) in the cyt *c* content was corrected by the expression of CcmE^{His}. **B** Western blotting against the His tag of the recombinant CcmE.
